# Surgical Human Resources According to Types of Health Care Facility: An Assessment in Low- and Middle-Income Countries

**DOI:** 10.1007/s00268-017-4078-4

**Published:** 2017-06-12

**Authors:** Shirwa Sheik Ali, Zahra Jaffry, Meena N. Cherian, Teena Kunjumen, Annette M. Nkwowane, Andrew J. M. Leather, Hernan Montenegro Von Muhlenbrock, Edward Kelley, James Campbell

**Affiliations:** 1 Academic Centre, Shepard’s House, Guy’s Campus, Kings’s College London School of Medicine, SE1 1UL London, England, UK; 2 Global Initiative for Emergency and Essential Surgical Care, Service Delivery and Safety Department, World Health Organization, Geneva, Switzerland; 3 Human Resources for Health Department, World Health Organization, Geneva, Switzerland; 4 King’s Centre for Global Health, King’s Health Partners and King’s College London, London, UK; 5 Health Workforce Alliance, World Health Organization, Geneva, Switzerland

## Abstract

**Background:**

A robust health care system providing safe surgical care to a population can only be achieved in conjunction with access to competent surgical personnel. It has been reported that 5 billion people do not have access to safe, affordable surgical and anaesthesia care when needed. This study aims to fill the existing gap in evidence by quantifying shortfalls in trained personnel delivering safe surgical and anaesthetic care in low- and middle-income countries (LMICs) according to the type of health care facility.

**Methods:**

We conducted secondary analysis of 1323 health facilities, in 35 low- and middle-income countries using facility-based cross-sectional data from the World Health Organization Situational Analysis Tool to Assess Emergency and Essential Surgical Care.

**Results:**

The majority of surgical and anaesthetic care in LMICs was provided by general doctors (range 13.8–41.1%; mean 27.1%). Non-physicians made up a significant proportion of the surgical workforce in LMICs. 26.76% of the surgical and anaesthetic workforce was provided by clinical medical officers and nurses. Private/NGO/mission hospitals, large, well-resourced institutions had the highest proportion of surgeons compared to any other type of health care facility at 27.92%. This compares to figures of 18.2 and 19.96% of surgeons at health centres and subdistrict/community hospitals, respectively, representing the lowest level of health facility.

**Conclusions:**

We highlight the significant proportion of non-physicians delivering surgical and anaesthetic care in LMICs and illustrate wide variations according to the type of health care facility.

## Introduction

A robust health care system providing safe surgical care to a population can only be achieved in conjunction with access to competent surgical personnel. It has been reported that the poorest third of the world’s population obtain only 3.5% of surgical operations conducted globally and that 5 billion people do not have access to safe, affordable surgical and anaesthesia care when needed [1, 2]. This is, in part, due to shortfalls in trained personnel, infrastructure and political priority [3, 4].

There is a common misconception that improving access to safe surgical and anaesthetic care in low- and middle-income countries (LMICs) is too expensive. However, multiple studies dismiss this notion, demonstrating the significant cost-effectiveness of surgical interventions in LMICs when compared to standard national health interventions [5, 6] and call for its acknowledgement as a critical component of the post-2015 global health agenda [7, 8].

The World Health Organization (WHO) launched the Global Initiative for Emergency and Essential Surgical Care (GIEESC) in December 2005: a global forum convening stakeholders representing health authorities, public health experts, non-governmental organizations (NGOs), civil and professional societies and individuals collaborating towards improving access to safe surgical and anaesthetic care in a global setting [9]. In 2007, GIEESC members developed the standardized WHO Situational Analysis Tool (SAT): a cross-sectional survey form used as an evidence-based tool to quantify surgical and anaesthetic capacity within participating facilities in LMICs. The SAT has been validated for assessing surgical capacity from various levels of health care facilities in LMICs and has been used to collect data from 55 LMICs from December 2007 through the present [10].

This study focuses on filling the existing gap in evidence by quantifying shortfalls in trained personnel delivering safe surgical and anaesthetic care in LMICs, using the WHO Situational Analysis Tool. We aim to describe these shortfalls according to various levels of health care facility, namely health centres, subdistrict/community hospitals, district/rural hospitals, general hospitals, provincial hospitals and private/non-governmental organization (NGO)/mission hospitals.

## Materials and methods

### Data collection

The standardized WHO Situational Analysis Tool (SAT) to assess access to emergency and essential surgical care was developed by the WHO Global Initiative for Essential and Emergency Surgical Care research group in November 2007. The WHO SAT includes 108 data points addressing four core sections: (1) infrastructure and health facility demographics; (2) health care personnel; (3) availability of surgical interventions; and (4) availability of surgical equipment and supplies. The availability of surgical equipment and supplies is based on the WHO Essential and Emergency Equipment List.

Data were collected by Ministries of Health, WHO country offices and by Global Initiative for Essential and Emergency Surgical Care (GIEESC) representatives in individual countries visiting the health facilities. These data were entered into the WHO EESC global database at the WHO headquarters in Geneva, Switzerland, from December 2007 through the present. However, only data entered into the database between December 2007 and August 2014 were included for the purposes of this paper.

### Data analysis

Countries providing assessments on less than 3 health care facilities were excluded from the aggregated data. This was in line with previous studies employing the WHO tool [3]. Health care facilities with incomplete data points for “Introduction” in section (infrastructure) and “Materials and methods” in section (human resources) of the WHO SAT were excluded.

Health care facilities included health centres, subdistrict/community hospitals, district/rural hospitals, general hospitals, provincial hospitals and private/non-governmental organization (NGO)/mission hospitals.

Ethical approval was deemed not necessary to be obtained for this study, as patient information was not included.

## Results

**Fig. 1:**
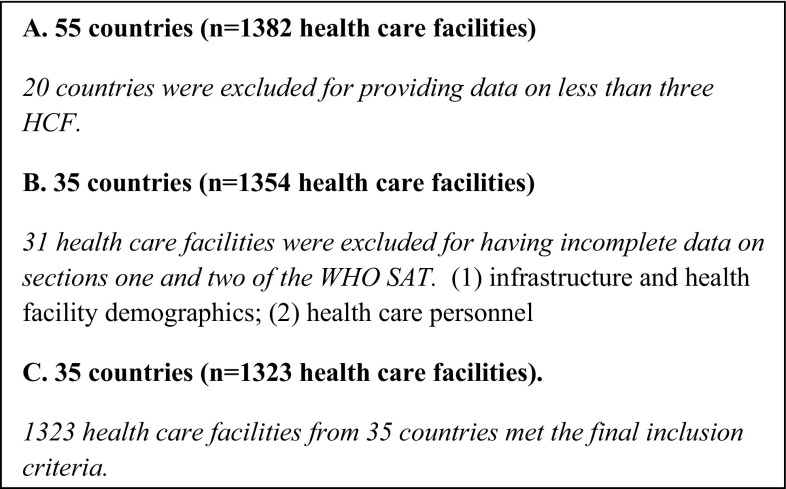
Study flow chart

All entries from the WHO SAT database are listed below with the number of health care facilities completing the SAT (Table 1). Those highlighted in green are LMICs providing assessments on less than 3 health care facilities and were, therefore, excluded from the aggregated data. There were a total of 1323 health care facilities from 35 countries which met the inclusion criteria (Fig. 1; Table 2).

**Table 1:** All 1382 health care facility entries from the WHO SAT database

No.	Country	No. facilities completing a survey
1	Afghanistan	26
2	Argentina	9
3	Bangladesh	267
4	Bhutan	1
5	Botswana	1
6	Burindi	2
7	Burkina Faso	2
8	Cambodia	1
9	Cameroon	3
10	Chad	3
11	China	8
12	Democratic Republic of the Congo	19
13	Egypt	1
14	Ethiopia	23
15	Fiji	2
16	Gabon	1
17	Gambia	75
18	Ghana	22
19	Haiti	54
20	Honduras	1
21	India	172
22	Indonesia	4
23	Kenya	129
24	Liberia	24
25	Libyan Arab Jamahiriya	1
26	Madagascar	2
27	Malawi	19
28	Maldives	1
29	Mali	3
30	Mongolia	43
31	Mozambique	4
32	Myanmar	20
33	Nicaragua	2
34	Niger	21
35	Nigeria	123
36	Pakistan	10
37	Papua New Guinea	25
38	Peru	2
39	Puerto Rico	1
40	Rwanda	3
41	Saint Lucia	1
42	Sao Tome and Principe	5
43	Sierra Leone	12
44	Solomon Islands	10
45	Somalia	14
46	Sri Lanka	39
47	Sudan	2
48	Togo	1
49	Trinidad and Tobago	54
50	Uganda	38
51	United Republic of Tanzania	49
52	Venezuela	2
53	Viet Nam	19
54	Zambia	5
55	Zimbabwe	1
	TOTAL	1382

**Table 2:** Characteristics of countries included in study according to types of type of health care facility

No.	Country	LIC/MIC*	No. facilities completing a survey	No. facilities included	No. health centres	No. SD/community hospital	No. district/rural hospital	No. provincial hospital	No. general hospital	No. private/NGO/mission hospital	Per cent of data
1	Afghanistan	LIC	26	25	1	0	9	4	10	1	1.89
2	Argentina	MIC	9	9	1	0	3	3	1	1	0.68
3	Bangladesh	LIC	267	263	6	218	23	0	16	0	19.89
4	Cameroon	MIC	3	3	0	0	0	3	0	0	0.23
5	Chad	LIC	3	3	0	0	2	0	1	0	0.23
6	China	MIC	8	8	4	0	1	2	1	0	0.6
7	Democratic Republic of the Congo	LIC	19	19	0	0	5	1	8	5	1.44
8	Ethiopia	LIC	23	23	0	0	8	0	10	5	1.74
9	Gambia	LIC	75	74	53	0	5	1	4	11	5.59
10	Ghana	MIC	22	22	0	0	15	0	1	6	1.66
11	Haiti	LIC	54	54	1	0	16	7	7	23	4.08
12	India	MIC	172	168	77	1	58	3	6	23	12.7
13	Indonesia	MIC	4	4	2	0	1	0	1	0	0.3
14	Kenya	LIC	129	128	58	3	38	10	4	15	9.67
15	Liberia	LIC	24	24	4	0	7	8	1	4	1.81
16	Malawi	LIC	19	19	0	0	13	1	1	4	1.44
17	Mali	LIC	3	3	0	0	0	0	3	0	0.23
18	Mongolia	MIC	43	34	2	0	15	13	2	2	2.57
19	Mozambique	LIC	4	4	1	0	3	0	0	0	0.3
20	Myanmar	LIC	20	20	0	0	6	1	13	0	1.51
21	Niger	LIC	21	20	0	0	13	3	3	1	1.51
22	Nigeria	MIC	123	123	3	0	0	5	17	98	9.3
23	Pakistan	MIC	10	5	1	0	1	0	0	3	0.38
24	Papua New Guinea	MIC	25	24	6	0	12	1	3	2	1.81
25	Rwanda	LIC	3	3	0	0	3	0	0	0	0.23
26	Sao Tome and Principe	MIC	5	5	3	0	1	0	1	0	0.38
27	Sierra Leone	LIC	12	11	0	0	5	2	3	1	0.83
28	Solomon Islands	MIC	10	10	0	0	0	4	1	5	0.76
29	Somalia	LIC	14	14	1	0	0	5	3	5	1.06
30	Sri Lanka	MIC	39	36	9	0	21	0	6	0	2.72
31	Trinidad and Tobago	MIC	54	54	50	1	2	0	1	0	4.08
32	Uganda	LIC	38	38	23	0	1	3	2	9	2.87
33	United Republic of Tanzania	LIC	49	49	11	0	20	5	5	8	3.7
34	Vietnam	MIC	19	19	1	0	17	1	0	0	1.44
35	Zambia	MIC	5	5	0	0	1	1	2	1	0.38
		TOTAL	1354	1323	318	223	325	87	137	233	100%

* As defined by the World Bank Classification System based on 2012 GNI per capita with LIC making $1025 or less, and MIC making $1026-$12,475

*SD* subdistrict hospital; *NGO non*-*governmental organization*

### Types of health care facility

**Fig. 2:**
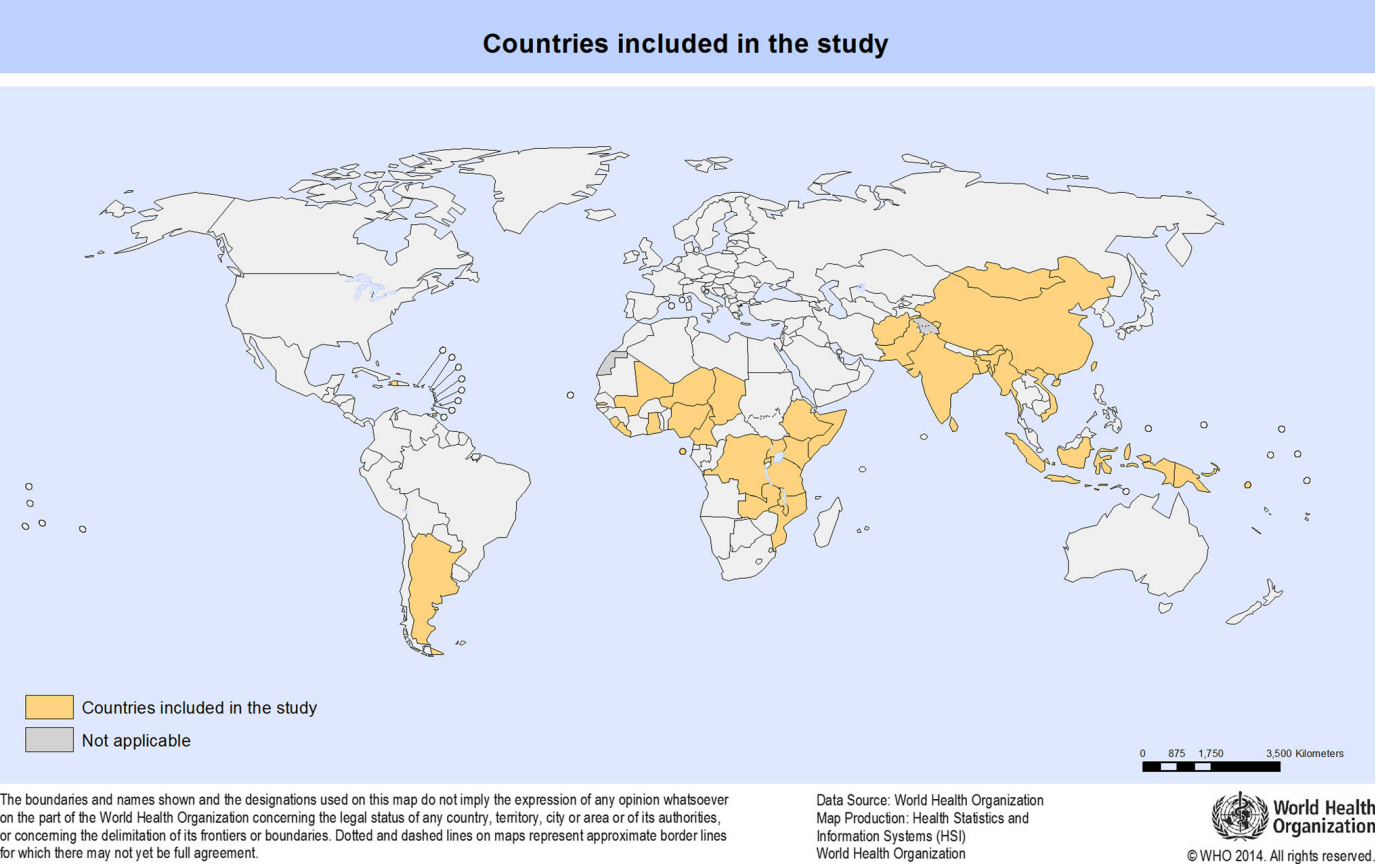
35 LMICs included in the final study

**Fig. 3:**
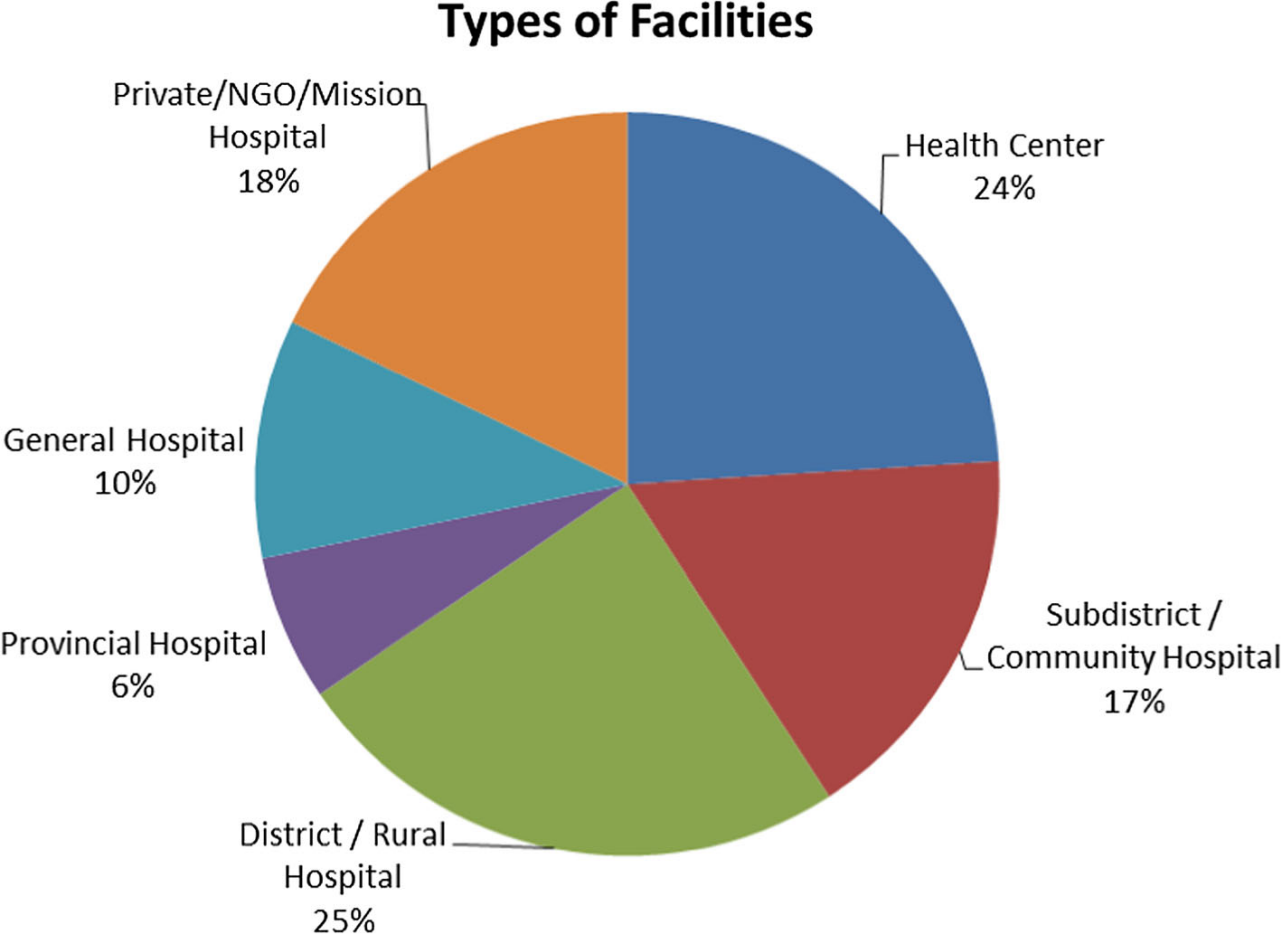
Of 1323 facilities included in analysis, types of facilities by percentage of total

There were a total of 1323 facilities surveyed from 35 LMICs (Fig. 2). The majority of facilities were district/rural hospitals (24.6%), followed by health centres (24%), private/NGO/mission hospitals (17.8%), subdistrict/community hospitals (16.7%), general hospitals (10.4%) and provincial hospitals (6.4%) as shown in Fig. 3.

### Personnel

**Fig. 4:**
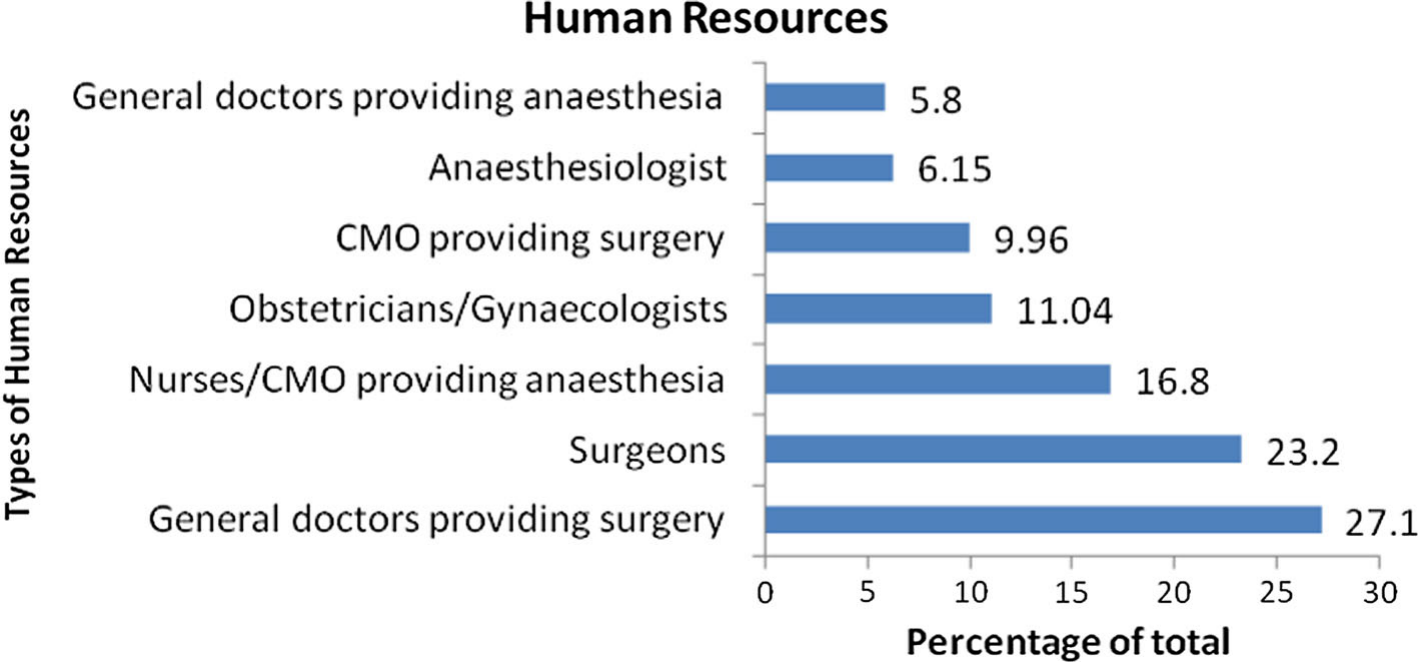
Of 1323 facilities included in analysis, types of human resources by percentage of total

To assess shortfalls in trained personnel delivering surgical and anaesthetic care in LMICs, we looked at the different types of human resources present across all types of health care facility included in analysis (Fig. 4). General doctors providing surgery constituted the bulk of trained personnel providing surgical and anaesthetic care across all types of health care facility (27.1%), followed by surgeons (23.2%), nurses/clinical medical officers (CMO) providing anaesthesia (16.8%), obstetricians/gynaecologists (11.04%), clinical medical officers providing surgery (10%), anaesthesiologists (6.2%) and finally general doctors providing anaesthesia (5.8%).

### Human resources according to types of health care facility

**Fig. 5:**
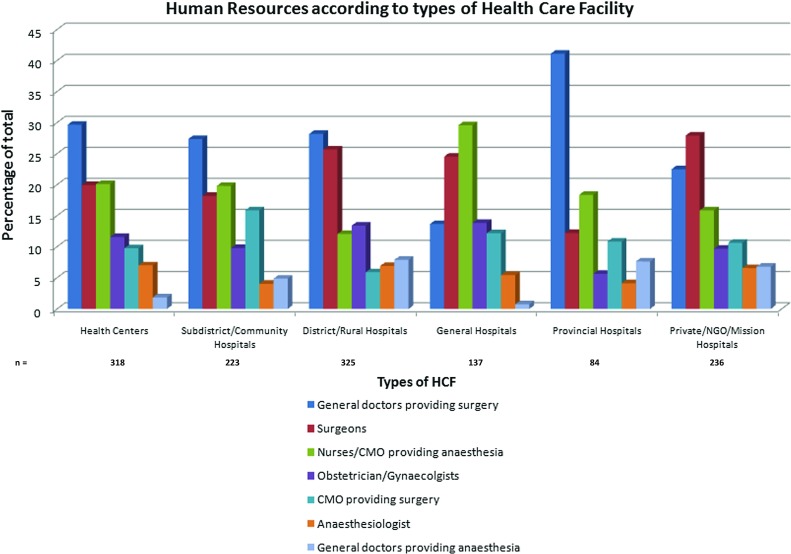
Human resources according to types of health care facility

From a total of 1323 health care facilities included in analysis, the bulk of personnel providing surgical and anaesthetic care were general doctors providing surgery (range 13.8–41.1%; mean 27.1%), surgeons (range 12.21–27.9%; mean 23.2%) and nurses/clinical medical officers providing anaesthesia (range 12.1–29.6%; mean 16.8%) (Figs. 4, 5).

This majority of personnel providing surgical and anaesthetic care varied considerably according to the type of health care facility. Health centres, representing the lowest level of health care facility, had surgeons representing 20% of their human resources, compared to a figure of 27.9% at private/NGO/mission hospitals: typically well-equipped institutions (Fig. 5).

## Discussion

Our analysis demonstrates that the majority of surgical and anaesthetic care in LMICs is provided by general doctors (range 13.8–41.1%; mean 27.1%). However, the team providing such care is highly varied, with surgeons, nurses, clinical medical officers (CMOs), obstetricians/gynaecologists and anaesthesiologists making significant contributions to the surgical and anaesthetic team also. If we combine the proportion of CMOs providing surgery with nurses/CMOs providing anaesthesia, this figure stands at 26.76%. Therefore, non-physicians make up a significant proportion of the surgical workforce in LMICs.

Shortage of surgical staff in LMICs has partly been addressed through international agencies and programmes run by local or expatriate surgeons [11]. This is reflected in the data for private/NGO/mission hospitals: large, well-resourced institutions with the highest proportion of surgeons compared to any other type of health care facility at 27.92% (Fig. 5). This compares to figures of 18.2 and 19.96% of surgeons at health centres and subdistrict/community hospitals, respectively, representing the lowest level of health facility (Fig. 5).

The International Classification of Health Workers (ICHW) has indicated that certain non-surgical personnel, including general medical practitioners and nursing professionals, have the scope to carry out certain surgical procedures within their role [12]. Programmes to train such personnel in surgical procedures, such as caesarean section and abscess drainage, have been adopted in certain countries, including Tanzania, Malawi and the Democratic Republic of Congo [11]. Compared with physician programmes, these can be highly cost-effective, have favourable outcomes and have better recruitment and retention of staff [13]. To ensure concerns about the quality and safety of care are allayed, standardised competencies and training programmes for non-physicians providing surgical and anaesthetic care need to be established.

A critical step in helping to define scalable solutions for the provision of quality surgical and anaesthesia care has been the recent launch of the Lancet Commission on Global Surgery (LCoGS). A study conducted by the LCoGS analysed national data from WHO member countries on the number of specialist surgeons, anaesthetists and obstetricians (SAOs) per 100,000 population and its correlation with the number of maternal deaths per 100,000 live births [14]. From this, the LCoGS introduced a surgical preparedness metric, suggesting a target for a global workforce of SAOs to be set between 20 and 40 per 100,000 of a population in order to provide the world’s missing surgical procedures [14]. How this target relates to non-specialist surgical providers is unclear. This study aims to fill in the existing gap in evidence by highlighting the significant proportion of non-physicians providing surgical and anaesthetic care in LMICs.

This study has several limitations. The WHO Situational Analysis Tool database represents a sample of convenience and is therefore susceptible to selection bias. The health care facilities in the data are not necessarily geographically or demographically representative of their country. Furthermore, although the number of health care personnel at each health care facility is available, how they relate to care of patients is unclear. The data points collected from the WHO SAT are unable to differentiate which kind of physicians provided surgical or anaesthetic care. A further study could disaggregate this further, demonstrating what kinds of physicians provide care in these categories.

It is important to note that there is a wide variety of health care structures across LMICs. The data collected in this study focussed more on health worker count than health systems. A potential area for future research would separate LMICs to examine whether there are different perspectives in different parts of the world with regards to non-physician providers administering surgical and/or anaesthetic care.

We highlight the significant proportion of non-physicians delivering surgical and anaesthetic care in LMICs and illustrate wide variations according to the type of health care facility.
